# (*RS*)-(2-Bromo-4-fluoro­anilino)[2-(4,6-dimethoxy­pyrimidin-2-yl­oxy)phen­yl]acetonitrile

**DOI:** 10.1107/S1600536809033819

**Published:** 2009-08-29

**Authors:** Yuan-xiang Li

**Affiliations:** a Chemistry and Chemical Engineering Department, Huaihua University, Huaihua 418008, People’s Republic of China

## Abstract

In the title compound, C_20_H_16_BrFN_4_O_3_, the pyrimidine and 2-bromo-4-fluoro­phenyl rings are twisted away from the central benzene ring, making dihedral angles of 77.7 (1) and 85.5 (1), respectively. A pair of C—H⋯F inter­actions is involved in an *R*_2_^2^(8) motif, linking the mol­ecules into dimers. These ring motifs are situated about the crystallographic centres of symmetry. C—H⋯O hydrogen bonds link the dimers into chains running parallel to [1
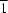
1]. Additionally, a weak C—F⋯π-electron ring inter­action was observed in the crystal packing [F⋯*Cg* = 3.459 (4) Å; *Cg* is the centroid of the pyrimidine ring]. There is also an intra­molecular N—H⋯Br inter­action in the structure.

## Related literature

Pyrimidinylbenzoates are highly effective herbicides with acetohydroxy­acid synthase (AHAS) as a target, see: Duggleby & Pang (2000 ▶). For related structures, see: Li & Huang (2007 ▶); Li & Wang (2007 ▶). For bond-length data, see: Allen *et al.* (1987 ▶). For graph-set motifs, see: Etter *et al.* (1990 ▶).
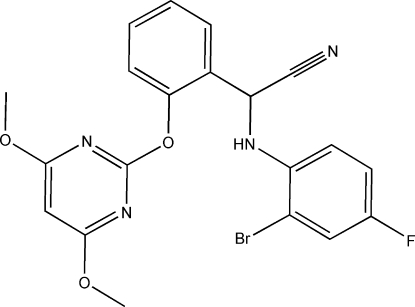


## Experimental

### 

#### Crystal data

C_20_H_16_BrFN_4_O_3_
*M* *_r_* = 459.28
Triclinic, 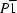

*a* = 8.9131 (4) Å
*b* = 10.6646 (4) Å
*c* = 11.7804 (5) Å
α = 67.235 (1)°
β = 82.088 (1)°
γ = 81.277 (2)°
*V* = 1016.77 (7) Å^3^
*Z* = 2
Mo *K*α radiation
μ = 2.06 mm^−1^
*T* = 299 K
0.25 × 0.20 × 0.20 mm

#### Data collection

Bruker SMART APEX CCD area-detector diffractometer
Absorption correction: multi-scan (*SADABS*; Sheldrick, 1996 ▶) *T*_min_ = 0.606, *T*_max_ = 0.662
10737 measured reflections
4588 independent reflections
2636 reflections with *I* > 2σ(*I*)
*R*_int_ = 0.027

#### Refinement

*R*[*F* ^2^ > 2σ(*F* ^2^)] = 0.055
*wR*(*F* ^2^) = 0.166
*S* = 1.05
4588 reflections
267 parameters
1 restraint
H atoms treated by a mixture of independent and constrained refinement
Δρ_max_ = 0.83 e Å^−3^
Δρ_min_ = −0.47 e Å^−3^

### 

Data collection: *SMART* (Bruker, 2001 ▶); cell refinement: *SAINT-Plus* (Bruker, 2001 ▶); data reduction: *SAINT-Plus*; program(s) used to solve structure: *SHELXS97* (Sheldrick, 2008 ▶); program(s) used to refine structure: *SHELXL97* (Sheldrick, 2008 ▶); molecular graphics: *PLATON* (Spek, 2009 ▶); software used to prepare material for publication: *PLATON*.

## Supplementary Material

Crystal structure: contains datablocks global, I. DOI: 10.1107/S1600536809033819/fb2161sup1.cif

Structure factors: contains datablocks I. DOI: 10.1107/S1600536809033819/fb2161Isup2.hkl

Additional supplementary materials:  crystallographic information; 3D view; checkCIF report

## Figures and Tables

**Table 1:** Hydrogen-bond geometry (Å, °)

*D*—H⋯*A*	*D*—H	H⋯*A*	*D*⋯*A*	*D*—H⋯*A*
N4—H4*A*⋯Br1	0.830 (18)	2.74 (3)	3.065 (2)	105 (3)
C13—H13⋯O3	0.98	2.41	2.763 (3)	101
C17—H17⋯F1^i^	0.93	2.61	3.541 (4)	175
C20—H20⋯O3^ii^	0.93	2.68	3.477 (4)	145
C13—H13⋯O3^ii^	0.98	2.53	3.433 (3)	153

Symmetry codes: (i) 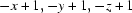; (ii) 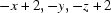.

**Fig. 1.**
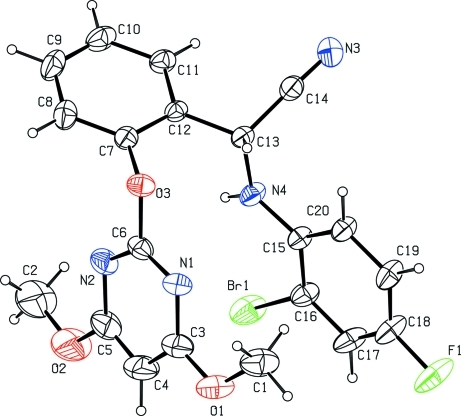
The title molecule with the atom-numbering scheme. The displacement ellipsoids are drawn at the 30% probability level.

**Fig. 2.**
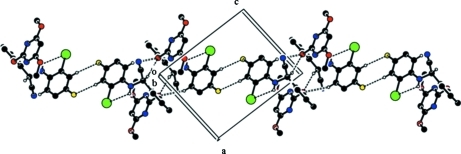
Section of the title structure, showing the a chain of the molecules linked by weak C-H···F and C-H···O interactions represented by dashed lines (Tab. 1). The chain is parallel to [111]. Also the N-H···Br interaction is shown. For the sake of clarity, the H atoms not involved in the hydrogen-bonds pattern have been omitted. Color code: C, black; H, white; N, blue; O, red; F, yellow; Br, green.

## supplementary crystallographic information

### Comment

Pyrimidine derivatives are rich in various biological properties. In particular, pyrimidinylbenzoates are highly effective herbicides with acetohydroxyacid synthase (AHAS) as a target (Duggleby & Pang, 2000). Here we report the crystal structure of one of such a pyrimidine derivative.

In the title molecule (Fig. 1), the bond lengths and angles are within normal ranges (Allen *et al.*, 1987) being in accordance with the corresponding values in similar compounds (Li & Huang, 2007; Li & Wang, 2007). The pyrimidine (N1//N2//C3—C6) and the benzene rings (C15—C20) are twisted away from the mid-benzene ring (C7—C12) with the dihedral angles equal to 77.7 (1) and 85.4 (1)°, respectively. The interplanar angle between the pyrimidine (N1//N2//C3—C6) and the benzene ring (C15—C20) is only 16.6 (1)°.

Pairs of C—H···F interactions form a graph-set R_2_^2^(8) (Etter *et al.*, 1990) about the crystallographic centres of symmetry, *i. e.*  the molecules form dimers. C-H···O hydrogen bonds link these dimers into one-dimensional chains running parallel to the [111] direction (Fig. 2). Additionally, a weak C—F···π-electron ring interaction is present in the crystal structure (F···Cg = 3.459 (4) Å, C18···Cg = 3.779 (5) Å, C···F···Cg = 92.9 (3)°. (Cg is the centroid of the pyrimidine ring defined by the atoms N1//N2//C3—C6; symmetry code *x*-1, *y*, *z*)). Moreover, there is an intramolecular N-H···Br interaction in the structure (Tab. 1; Fig. 2)).

### Experimental

Solution of 2-(4,6-dimethoxypyrimidin-2-yloxy)benzaldehyde (0.26 g, 1 mmol) and 4-bromo-2-fluoroaniline (0.21 g, 1.1 mmol) in 10 ml of methanol was stirred at room temperature for 0.5 h. Then trimethylsilanecarbonitrile (TMSCN) (0.15 g, 1.5 mmol) was added and the reaction mixture was stirred for another 12 h. The resulting mixture was filtered off by suction and the filter cake was washed with little methanol. The crude product was dried under infrared lamp and the title compound was obtained as a white solid (the yield 0.22 g, 74%, melting point: 383–385 K). The product was recrystallized from ethanol at room temperature to give block colourless crystals with average size: 0.20 × 0.15 × 0.10 mm.

### Refinement

All the H atoms could have been determined in the difference electron density map. The N4—H4A distance was restrained to 0.86 (1) Å while the displacement parameter of H4A this atom was constrained: *U*_iso_(H4A) = 1.2*U*_eq_(N4). The remaining H atoms were positioned into idealized positions, with C—H = 0.93, 0.98 and 0.96 Å for aryl, methine and methyl groups, respectively. *U*_iso_(H_aryl_/_methine_) = 1.2*U*_eq_(C_aryl_/_methine_) and *U*_iso_(H_methyl_) = 1.5*U*_eq_(C_methyl_).

### Crystal data

**Table:**

C_20_H_16_BrFN_4_O_3_	*Z* = 2
*M**_r_* = 459.28	*F*(000) = 464
Triclinic, *P*1	*D*_x_ = 1.500 Mg m^−^^3^
Hall symbol: -P 1	Melting point = 383–385 K
*a* = 8.9131 (4) Å	Mo *K*α radiation, λ = 0.71073 Å
*b* = 10.6646 (4) Å	Cell parameters from 2760 reflections
*c* = 11.7804 (5) Å	θ = 2.3–24.1°
α = 67.235 (1)°	µ = 2.06 mm^−^^1^
β = 82.088 (1)°	*T* = 299 K
γ = 81.277 (2)°	Block, colourless
*V* = 1016.77 (7) Å^3^	0.25 × 0.20 × 0.20 mm

### Data collection

**Table:**

Bruker SMART APEX CCD area-detector diffractometer	4588 independent reflections
Radiation source: fine focus sealed Siemens Mo tube	2636 reflections with *I* > 2σ(*I*)
graphite	*R*_int_ = 0.027
0.3° wide ω scans	θ_max_ = 27.5°, θ_min_ = 2.1°
Absorption correction: multi-scan (*SADABS*; Sheldrick, 1996)	*h* = −11→11
*T*_min_ = 0.606, *T*_max_ = 0.662	*k* = −13→13
10737 measured reflections	*l* = −15→15

### Refinement

**Table:**

Refinement on *F*^2^	Primary atom site location: structure-invariant direct methods
Least-squares matrix: full	Secondary atom site location: difference Fourier map
*R*[*F*^2^ > 2σ(*F*^2^)] = 0.055	Hydrogen site location: difference Fourier map
*wR*(*F*^2^) = 0.166	H atoms treated by a mixture of independent and constrained refinement
*S* = 1.05	*w* = 1/[σ^2^(*F*_o_^2^) + (0.0884*P*)^2^ + 0.0427*P*] where *P* = (*F*_o_^2^ + 2*F*_c_^2^)/3
4588 reflections	(Δ/σ)_max_ < 0.001
267 parameters	Δρ_max_ = 0.83 e Å^−^^3^
1 restraint	Δρ_min_ = −0.46 e Å^−^^3^
59 constraints	

### Special details

**Table:**

Geometry. All esds (except the esd in the dihedral angle between two l.s. planes) are estimated using the full covariance matrix. The cell esds are taken into account individually in the estimation of esds in distances, angles and torsion angles; correlations between esds in cell parameters are only used when they are defined by crystal symmetry. An approximate (isotropic) treatment of cell esds is used for estimating esds involving l.s. planes.
Refinement. Refinement of F^2^ against ALL reflections. The weighted R-factor wR and goodness of fit S are based on F^2^, conventional R-factors R are based on F, with F set to zero for negative F^2^. The threshold expression of F^2^ > 2sigma(F^2^) is used only for calculating R-factors(gt) etc. and is not relevant to the choice of reflections for refinement. R-factors based on F^2^ are statistically about twice as large as those based on F, and R- factors based on ALL data will be even larger.

### Fractional atomic coordinates and isotropic or equivalent isotropic displacement parameters (Å^2^)

**Table:**

	*x*	*y*	*z*	*U*_iso_*/*U*_eq_	
Br1	0.87179 (6)	0.19137 (4)	0.47691 (3)	0.1134 (3)	
C1	0.8782 (7)	0.4408 (4)	0.8262 (5)	0.1187 (16)	
H1A	0.8212	0.3653	0.8427	0.178*	
H1B	0.8092	0.5218	0.8175	0.178*	
H1C	0.9392	0.4212	0.8935	0.178*	
C2	1.3827 (9)	0.1531 (8)	0.4604 (6)	0.174 (3)	
H2A	1.4643	0.1246	0.5134	0.261*	
H2B	1.4241	0.1733	0.3764	0.261*	
H2C	1.3183	0.0810	0.4843	0.261*	
C3	1.0640 (5)	0.3549 (4)	0.7042 (4)	0.0895 (11)	
C4	1.1396 (6)	0.3699 (5)	0.5916 (4)	0.1173 (15)	
H4	1.1326	0.4534	0.5254	0.141*	
C5	1.2246 (5)	0.2596 (5)	0.5801 (4)	0.1054 (14)	
C6	1.1638 (4)	0.1373 (3)	0.7778 (3)	0.0650 (8)	
C7	1.2094 (3)	−0.1058 (3)	0.8622 (2)	0.0581 (7)	
C8	1.3569 (4)	−0.1646 (4)	0.8608 (3)	0.0783 (9)	
H8	1.4359	−0.1196	0.8658	0.094*	
C9	1.3867 (4)	−0.2903 (4)	0.8520 (4)	0.0888 (11)	
H9	1.4867	−0.3307	0.8506	0.107*	
C10	1.2715 (5)	−0.3569 (4)	0.8453 (3)	0.0848 (11)	
H10	1.2933	−0.4422	0.8391	0.102*	
C11	1.1221 (4)	−0.2986 (3)	0.8477 (3)	0.0672 (8)	
H11	1.0439	−0.3444	0.8426	0.081*	
C12	1.0890 (3)	−0.1719 (3)	0.8578 (2)	0.0523 (6)	
C13	0.9288 (3)	−0.1037 (3)	0.8654 (2)	0.0537 (7)	
H13	0.9238	−0.0567	0.9230	0.064*	
C14	0.8165 (4)	−0.2064 (4)	0.9153 (3)	0.0695 (8)	
C15	0.7744 (3)	0.1025 (3)	0.7335 (3)	0.0580 (7)	
C16	0.7497 (4)	0.2024 (3)	0.6176 (3)	0.0721 (8)	
C17	0.6375 (5)	0.3112 (4)	0.6020 (4)	0.0936 (12)	
H17	0.6229	0.3771	0.5237	0.112*	
C18	0.5498 (5)	0.3204 (4)	0.7017 (4)	0.0931 (12)	
C19	0.5653 (4)	0.2244 (4)	0.8178 (4)	0.0841 (10)	
H19	0.5017	0.2317	0.8851	0.101*	
C20	0.6787 (4)	0.1154 (3)	0.8330 (3)	0.0689 (8)	
H20	0.6908	0.0496	0.9117	0.083*	
F1	0.4432 (4)	0.4297 (3)	0.6871 (3)	0.1400 (11)	
N1	1.0762 (3)	0.2366 (3)	0.8016 (2)	0.0702 (7)	
N2	1.2403 (3)	0.1355 (3)	0.6752 (3)	0.0813 (8)	
N3	0.7278 (4)	−0.2815 (4)	0.9507 (4)	0.1020 (11)	
N4	0.8911 (3)	−0.0024 (2)	0.7473 (2)	0.0667 (7)	
H4A	0.949 (3)	−0.018 (3)	0.692 (3)	0.080*	
O1	0.9737 (4)	0.4619 (3)	0.7162 (3)	0.1144 (10)	
O2	1.2974 (6)	0.2699 (5)	0.4710 (3)	0.1620 (17)	
O3	1.1745 (2)	0.0177 (2)	0.88013 (18)	0.0660 (5)	

### Atomic displacement parameters (Å^2^)

**Table:**

	*U*^11^	*U*^22^	*U*^33^	*U*^12^	*U*^13^	*U*^23^
Br1	0.1488 (5)	0.1099 (4)	0.0510 (3)	0.0276 (3)	−0.0032 (2)	−0.0139 (2)
C1	0.169 (5)	0.078 (3)	0.107 (4)	−0.005 (3)	−0.004 (3)	−0.037 (3)
C2	0.200 (7)	0.172 (6)	0.102 (4)	−0.011 (5)	0.069 (5)	−0.030 (4)
C3	0.111 (3)	0.070 (2)	0.075 (2)	−0.019 (2)	−0.012 (2)	−0.0079 (18)
C4	0.142 (4)	0.082 (3)	0.087 (3)	−0.011 (3)	−0.001 (3)	0.010 (2)
C5	0.107 (3)	0.117 (3)	0.061 (2)	−0.026 (3)	0.021 (2)	−0.004 (2)
C6	0.071 (2)	0.0700 (19)	0.0497 (16)	−0.0244 (16)	−0.0043 (14)	−0.0116 (14)
C7	0.0611 (19)	0.0630 (17)	0.0426 (14)	−0.0014 (14)	−0.0020 (12)	−0.0140 (12)
C8	0.059 (2)	0.101 (3)	0.069 (2)	0.0008 (18)	−0.0023 (15)	−0.0300 (19)
C9	0.065 (2)	0.107 (3)	0.077 (2)	0.026 (2)	−0.0007 (17)	−0.030 (2)
C10	0.102 (3)	0.0647 (19)	0.075 (2)	0.022 (2)	0.003 (2)	−0.0269 (17)
C11	0.077 (2)	0.0568 (16)	0.0649 (19)	0.0034 (15)	0.0013 (15)	−0.0256 (14)
C12	0.0566 (16)	0.0501 (14)	0.0428 (13)	0.0045 (12)	−0.0038 (11)	−0.0132 (11)
C13	0.0570 (16)	0.0505 (14)	0.0485 (14)	0.0048 (12)	−0.0048 (12)	−0.0170 (12)
C14	0.061 (2)	0.0687 (19)	0.072 (2)	0.0060 (17)	−0.0083 (16)	−0.0233 (16)
C15	0.0625 (17)	0.0557 (15)	0.0544 (16)	0.0062 (13)	−0.0135 (13)	−0.0209 (13)
C16	0.087 (2)	0.0671 (18)	0.0529 (17)	0.0105 (16)	−0.0157 (15)	−0.0164 (14)
C17	0.117 (3)	0.072 (2)	0.075 (2)	0.033 (2)	−0.036 (2)	−0.0161 (18)
C18	0.100 (3)	0.080 (2)	0.095 (3)	0.043 (2)	−0.034 (2)	−0.038 (2)
C19	0.076 (2)	0.094 (2)	0.080 (2)	0.0272 (18)	−0.0168 (18)	−0.041 (2)
C20	0.0686 (19)	0.0682 (18)	0.0601 (18)	0.0153 (15)	−0.0097 (14)	−0.0203 (15)
F1	0.155 (2)	0.1206 (19)	0.129 (2)	0.0875 (17)	−0.0487 (17)	−0.0542 (16)
N1	0.0928 (19)	0.0533 (14)	0.0608 (15)	−0.0158 (14)	−0.0069 (13)	−0.0143 (12)
N2	0.0848 (19)	0.0860 (19)	0.0580 (16)	−0.0188 (15)	0.0059 (14)	−0.0110 (14)
N3	0.070 (2)	0.094 (2)	0.132 (3)	−0.0117 (18)	−0.0006 (19)	−0.033 (2)
N4	0.0713 (16)	0.0654 (14)	0.0482 (14)	0.0183 (12)	−0.0040 (11)	−0.0146 (11)
O1	0.158 (3)	0.0604 (15)	0.103 (2)	−0.0037 (16)	−0.018 (2)	−0.0078 (14)
O2	0.190 (4)	0.146 (3)	0.082 (2)	−0.004 (3)	0.054 (2)	0.004 (2)
O3	0.0870 (14)	0.0585 (12)	0.0492 (11)	−0.0112 (10)	−0.0040 (10)	−0.0159 (9)

### Geometric parameters (Å, °)

**Table:**

Br1—C16	1.887 (3)	C9—C10	1.361 (6)
C1—O1	1.409 (5)	C9—H9	0.9300
C1—H1A	0.9600	C10—C11	1.383 (5)
C1—H1B	0.9600	C10—H10	0.9300
C1—H1C	0.9600	C11—C12	1.386 (4)
C2—O2	1.397 (8)	C11—H11	0.9300
C2—H2A	0.9600	C12—C13	1.508 (4)
C2—H2B	0.9600	C13—N4	1.439 (3)
C2—H2C	0.9600	C13—C14	1.491 (5)
C3—O1	1.335 (5)	C13—H13	0.9800
C3—N1	1.339 (4)	C14—N3	1.132 (4)
C3—C4	1.364 (6)	C15—N4	1.383 (4)
C4—C5	1.342 (7)	C15—C16	1.390 (4)
C4—H4	0.9300	C15—C20	1.391 (4)
C5—O2	1.330 (5)	C16—C17	1.383 (5)
C5—N2	1.365 (5)	C17—C18	1.347 (5)
C6—N2	1.310 (4)	C17—H17	0.9300
C6—N1	1.314 (4)	C18—F1	1.358 (4)
C6—O3	1.376 (3)	C18—C19	1.365 (5)
C7—C8	1.369 (4)	C19—C20	1.392 (5)
C7—C12	1.387 (4)	C19—H19	0.9300
C7—O3	1.396 (3)	C20—H20	0.9300
C8—C9	1.367 (5)	N4—H4A	0.830 (18)
C8—H8	0.9300		
			
O1—C1—H1A	109.5	C12—C11—H11	120.0
O1—C1—H1B	109.5	C11—C12—C7	118.1 (3)
H1A—C1—H1B	109.5	C11—C12—C13	123.2 (3)
O1—C1—H1C	109.5	C7—C12—C13	118.8 (2)
H1A—C1—H1C	109.5	N4—C13—C14	111.1 (2)
H1B—C1—H1C	109.5	N4—C13—C12	111.2 (2)
O2—C2—H2A	109.5	C14—C13—C12	111.2 (2)
O2—C2—H2B	109.5	N4—C13—H13	107.7
H2A—C2—H2B	109.5	C14—C13—H13	107.7
O2—C2—H2C	109.5	C12—C13—H13	107.7
H2A—C2—H2C	109.5	N3—C14—C13	177.8 (4)
H2B—C2—H2C	109.5	N4—C15—C16	120.6 (3)
O1—C3—N1	119.5 (4)	N4—C15—C20	122.5 (3)
O1—C3—C4	118.4 (4)	C16—C15—C20	116.8 (3)
N1—C3—C4	122.1 (4)	C17—C16—C15	121.7 (3)
C5—C4—C3	117.5 (4)	C17—C16—Br1	118.5 (3)
C5—C4—H4	121.3	C15—C16—Br1	119.8 (2)
C3—C4—H4	121.3	C18—C17—C16	119.1 (3)
O2—C5—C4	119.0 (4)	C18—C17—H17	120.5
O2—C5—N2	117.6 (5)	C16—C17—H17	120.5
C4—C5—N2	123.4 (4)	C17—C18—F1	119.1 (4)
N2—C6—N1	130.7 (3)	C17—C18—C19	122.3 (3)
N2—C6—O3	116.9 (3)	F1—C18—C19	118.5 (4)
N1—C6—O3	112.5 (3)	C18—C19—C20	118.3 (3)
C8—C7—C12	121.7 (3)	C18—C19—H19	120.9
C8—C7—O3	120.4 (3)	C20—C19—H19	120.9
C12—C7—O3	117.7 (2)	C15—C20—C19	121.7 (3)
C9—C8—C7	119.2 (3)	C15—C20—H20	119.2
C9—C8—H8	120.4	C19—C20—H20	119.2
C7—C8—H8	120.4	C6—N1—C3	114.1 (3)
C10—C9—C8	120.7 (3)	C6—N2—C5	112.2 (3)
C10—C9—H9	119.7	C15—N4—C13	123.3 (2)
C8—C9—H9	119.7	C15—N4—H4A	127 (3)
C9—C10—C11	120.5 (3)	C13—N4—H4A	110 (3)
C9—C10—H10	119.8	C3—O1—C1	118.3 (3)
C11—C10—H10	119.8	C5—O2—C2	118.1 (4)
C10—C11—C12	119.9 (3)	C6—O3—C7	118.4 (2)
C10—C11—H11	120.0		
			
O1—C3—C4—C5	−177.2 (5)	C16—C17—C18—C19	−1.4 (7)
N1—C3—C4—C5	2.0 (7)	C17—C18—C19—C20	1.7 (7)
C3—C4—C5—O2	178.7 (4)	F1—C18—C19—C20	−177.5 (4)
C3—C4—C5—N2	−1.0 (8)	N4—C15—C20—C19	177.7 (3)
C12—C7—C8—C9	1.6 (5)	C16—C15—C20—C19	−1.1 (5)
O3—C7—C8—C9	175.3 (3)	C18—C19—C20—C15	−0.3 (6)
C7—C8—C9—C10	−0.3 (5)	N2—C6—N1—C3	0.0 (5)
C8—C9—C10—C11	−0.2 (6)	O3—C6—N1—C3	179.6 (3)
C9—C10—C11—C12	−0.4 (5)	O1—C3—N1—C6	177.7 (3)
C10—C11—C12—C7	1.6 (4)	C4—C3—N1—C6	−1.4 (6)
C10—C11—C12—C13	−177.9 (3)	N1—C6—N2—C5	0.9 (5)
C8—C7—C12—C11	−2.2 (4)	O3—C6—N2—C5	−178.8 (3)
O3—C7—C12—C11	−176.1 (2)	O2—C5—N2—C6	−180.0 (4)
C8—C7—C12—C13	177.3 (3)	C4—C5—N2—C6	−0.3 (7)
O3—C7—C12—C13	3.4 (4)	C16—C15—N4—C13	175.8 (3)
C11—C12—C13—N4	−99.4 (3)	C20—C15—N4—C13	−2.9 (5)
C7—C12—C13—N4	81.2 (3)	C14—C13—N4—C15	75.8 (4)
C11—C12—C13—C14	25.0 (4)	C12—C13—N4—C15	−159.8 (3)
C7—C12—C13—C14	−154.5 (3)	N1—C3—O1—C1	−8.6 (6)
N4—C15—C16—C17	−177.5 (3)	C4—C3—O1—C1	170.5 (4)
C20—C15—C16—C17	1.3 (5)	C4—C5—O2—C2	−178.7 (6)
N4—C15—C16—Br1	2.3 (4)	N2—C5—O2—C2	1.0 (9)
C20—C15—C16—Br1	−178.9 (2)	N2—C6—O3—C7	−25.3 (4)
C15—C16—C17—C18	−0.1 (6)	N1—C6—O3—C7	155.0 (3)
Br1—C16—C17—C18	−179.9 (3)	C8—C7—O3—C6	94.9 (3)
C16—C17—C18—F1	177.8 (4)	C12—C7—O3—C6	−91.1 (3)

### Hydrogen-bond geometry (Å, °)

**Table:**

*D*—H···*A*	*D*—H	H···*A*	*D*···*A*	*D*—H···*A*
N4—H4A···Br1	0.83 (2)	2.74 (3)	3.065 (2)	105 (3)
C13—H13···O3	0.98	2.41	2.763 (3)	101
C17—H17···F1^i^	0.93	2.61	3.541 (4)	175
C20—H20···O3^ii^	0.93	2.68	3.477 (4)	145
C13—H13···O3^ii^	0.98	2.53	3.433 (3)	153

Symmetry codes: (i) −*x*+1, −*y*+1, −*z*+1; (ii) −*x*+2, −*y*, −*z*+2.
